# Direct and Indirect Somatic Embryogenesis Induction in *Camellia oleifera* Abel

**DOI:** 10.3389/fpls.2021.644389

**Published:** 2021-03-26

**Authors:** Ming Zhang, Aibin Wang, Mou Qin, Xuejing Qin, Shiwen Yang, Shuchai Su, Yongjiang Sun, Lingyun Zhang

**Affiliations:** ^1^Key Laboratory of Forest Silviculture and Conservation of the Ministry of Education, The College of Forestry, Beijing Forestry University, Beijing, China; ^2^Baise Forestry Bureau of Guangxi Zhuang Autonomous Region, Baise, China

**Keywords:** *Camellia oleifera*, somatic embryogenesis, explants, genotype, phytohormones

## Abstract

*Camellia oleifera* Abel. is an important woody oil species; however, the shortage of rapid and industrialized seedling culture is a large constraint on the development of the tea oil industry. Somatic embryogenesis (SE) is one of the main powerful biotechnological tools for plant mass regeneration, but the largely unknown SE in *C. oleifera* limits the scale production of clonal plants. In this study, we described a high-efficiency SE system via direct and indirect pathways in *C. oleifera* and investigated the effect of genotype, explant age and phytohormones on SE. In the direct pathway, somatic embryos were highly induced from immature seeds 220 days after full blossom, and the development of embryoids was achieved with a combination of 0.19 mg/L 2,4-dichlorophenoxyacetic acid (2,4-D) and 0.05 mg/L thidiazuron (TDZ). In the indirect pathway, embryogenic calli were induced from the same explants in medium containing 1.5 mg/L 2,4-D, while 0.75 mg/L 2,4-D treatment led to high proliferation rates for embryogenic calli. The addition of 0.19 mg/L 2,4-D alone stimulated the production of globular embryos while causing a 75% loss of the induction rate in the heart embryo stage. Upon transfer of the globular embryos to phytohormone-free medium, an optimal induction rate of 62.37% from globular embryos to cotyledonary embryos was obtained. These data suggest that the subsequent differentiation process after the globular embryo stage in ISE is more similar to an endogenous phytohormones-driven process. Mature embryos germinated to produce intact plantlets on half-strength MS basal medium with a regeneration rate of 63.67%. Histological analysis confirmed the vascular bundle isolation of embryoids from the mother tissue. We further studied the different varieties and found that there were no significant genotype differences for SE induction efficiency in *C. oleifera*. Thus, we established a high-efficiency induction system for direct and indirect somatic embryogenesis (ISE) in *C. oleifera* and regenerated intact plantlets via SE, not organogenesis. ISE has a more complicated induction and regulatory mechanism than direct somatic embryogenesis. The improved protocol of SE would benefit mass propagation and genetic manipulation in *C. oleifera*.

## Introduction

The tea oil plant (*Camellia oleifera* Abel.), an evergreen shrub or dungarunga, is a woody oil species worldwide. The tea-oil extracted from seeds is a very healthy edible oil that contains abundant unsaturated fatty acids, especially oleic acid, at up to 85% (Wang et al., 2012), comparable to the best olive oil in the world. Tea oil trees are widely grown in subtropical and warm hilly regions of China (Zhuang, 2008). In recent years, the worldwide demand for tea oil has rapidly expanded due to the trend of consuming healthier oils, and the agricultural area reached 4.367 million hm^2^ in 2018 in China (State Forestry Bureau, 2009). The large demand for *C. oleifera* seedlings is accordingly expanding with the rapid development of the tea oil industry and has far outstripped the supply of quality seedlings.

In China, approximately 73% of tea oil trees are planted with seed-germinated seedlings. Due to the long vegetative growth cycle, unstable yield and great genetic variation of seed offspring, seed-germinated seedlings easily result in low-yield trees. Recently, grafting techniques have been widely applied for low-yield tree transformation and seedling propagation (Ruan and Mopper, 2017). Since heterozygous seeds are primarily employed as rootstocks in the process of grafted *C. oleifera* seedling propagation, the poor growth consistency of the seedling is one of the major limiting factors for the supply of higher quality seedlings. Moreover, owing to the great consumption of seed rootstock and low efficiency, it is relatively difficult to provide a mass of sustainable seedlings for the *C. oleifera* industry by grafting propagation alone. Thus, it is essential and urgent to establish a more efficient and industrialized propagation technique system to raise *C. oleifera* seedlings.

In plants, somatic embryogenesis (SE) represents a powerful biotechnological tool for clonal regeneration, germplasm conservation, and genetic improvement (Guan et al., 2016). Somatic embryos are characterized by large numbers, rapid production, high-efficiency induced seedling formation, relatively stable genetic characteristics, and small variations in regenerated plants; therefore, the seedlings induced by somatic embryos are close to the natural state of their occurrence and development (Pais, 2019). To date, SE has been reported in a variety of plant species, such as chestnut, oil palm, and walnut (Tulecke and Mcgranahan, 1985; Thuzar et al., 2010; Lu et al., 2017), especially in species such as the Norway spruce and Sweetgum, in which seedlings induced by somatic embryos are widely applied in afforestation practices on a large scale and generate large gains (Wang et al., 2016; Tikkinen et al., 2019). The in-depth research on SE and the application of somatic embryo seedlings in these woody species contributed to the production of higher quality seedlings and factory-cultivated seedlings raised at lower-cost.

Two different types of somatic embryogenic routes are generally involved in plants: direct somatic embryogenesis (DSE) and indirect somatic embryogenesis (ISE; Sharp et al., 1980). In DSE, there is no dedifferentiation stage, and embryonic cell formation can be completed directly from the surface of explants, in which minimal genetic reprogramming is involved; in contrast, ISE is a multistep regeneration process including somatic embryo formation, maturation, and conversion that requires major reprogramming (Arnold et al., 2002). Compared to DSE, ISE has a higher propagation efficiency and is applied for a longer period of frozen storage technology, which can lay a solid foundation for industrialized seedling culture by somatic embryo induction. In species such as *Murraya koenigii* and *Mangifera indica* (Paul et al., 2011; Sánchez and Dallos, 2018), two types of somatic embryogenetic pathways have been successfully established. However, it is more difficult for woody plants to induce somatic embryos as a result of more frequent rough-shaped embryos and poor-quality somatic embryos (Nugent et al., 2001; Pinto et al., 2002). To date, only one type of somatic embryogenetic pathway has been reported in some species. For instance, the DSE pathway was established in *Minthostachys verticillata* (Bertero et al., 2020), and the ISE pathway was established in *Fraxinus mandshurica* (Liu et al., 2020). Meanwhile, the poor synchrony of somatic embryo development, coupled with the slow growth rate and long cycle of woody plants, has further hampered the commercial application of raising SE seedlings.

Somatic embryogenesis is usually influenced by multiple factors, such as genotype, explant type and age, and phytohormones (Merkle et al., 1998; Carneros et al., 2009; Bakhshaie et al., 2010; Capelo et al., 2010; Varis et al., 2018; Hapsoro et al., 2020). Generally, the ability of an explant to undergo SE is closely related to the age and type of explants. In *Arabidopsis*, several *in vitro* somatic embryo systems have been developed, including immature and mature zygotic embryos, shoot apices, and flower buds (Ikeda-Iwai et al., 2002; Kobayashi et al., 2010; Barbara et al., 2018). In *Sapindus mukorossi*, 6-day-old leaves were reported to be the most appropriate for somatic embryo induction (Singh et al., 2015). In *Eucalyptus camaldulensis*, 10-day-old cotyledons showed the highest callus induction rate (Prakash and Gurumurthi, 2010). Similarly, Lu et al. (2017) reported that immature embryos 45–54 days after initial flowering had the best induction efficiency in chestnut. Phytohormones are another key influencing factors for SE. Most plant species undergoing SE are dependent on the powerful action of auxin and cytokinin, especially 2,4-dichlorophenoxyacetic acid (2,4-D) which regulates and balances endogenous auxin levels (Illyas Ahmad et al., 2013; Mahendran and Bai, 2016; Keshvari et al., 2018).

In *C. oleifera*, several studies on SE have been reported (Yan and Chen, 1980; Zhang, 2005; Peng, 2008; Hu et al., 2014); however, systematic investigations and observations of the DSE and ISE pathways in *C. oleifera* remain largely undescribed, especially during developmental processes at the histological level, considering that vascular bundle isolation from the mother tissue is an important sign to distinguish SE from organogenesis (Horstman et al., 2017). In addition, whether the genotype or explant developmental stages influence SE in *C. oleifera* remains elusive. Therefore, this study aimed to establish a reliable and reproducible somatic embryo regeneration system for *C. oleifera*. The effects of genotypes, developmental stages of explants and phytohormones on regeneration and propagation rates were further elucidated. The developed technical system in *C. oleifera* would contribute to industrialized seedling production, genetic manipulation and breeding improvement.

## Materials and Methods

### Plant Material

**FIGURE 1:**
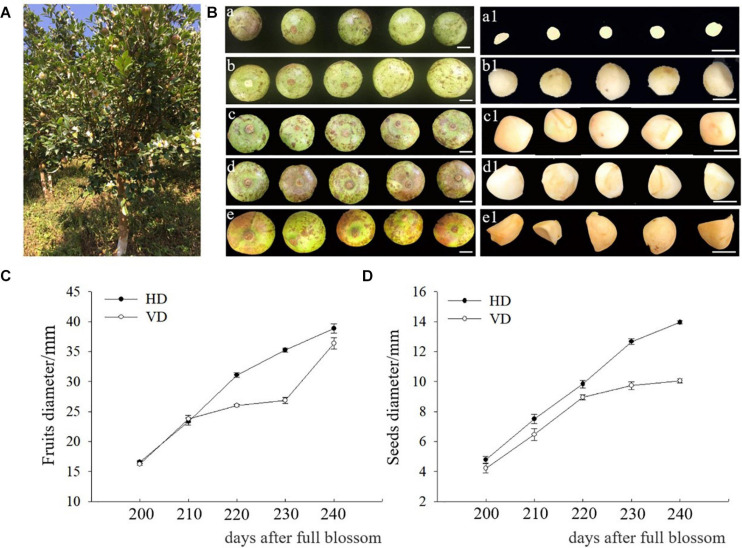
Sampling at different developmental stages of *C. oleifera*. **(A)** Mature tea-oil tree. **(B)** The morphology of fruits and seeds from different developmental stages. (a, a1) Fruits and seeds 200 days after full blossom. (b, b1) Fruits and seeds 210 days after full blossom. (c, c1) Fruits and seeds 220 days after full blossom. (d, d1) Fruits and seeds 230 days after full blossom. (e, e1) Fruits and seeds 240 days after full blossom. **(C)** Horizontal and vertical diameters (HD, VD) of fruits after full blossom. **(D)** Horizontal and vertical diameters of seeds after full blossom. Bars: (a, a1) = 6 mm, (b, b1) = 8 mm, (c, c1) = 10 mm, (d, d1) = 12 mm, and (e, e1) = 13 mm.

Seeds of *C. oleifera* cv. The “Cenruan 2” and “3 Hua” series, namely “Huashuo,” “Huaxin,” and “Huajin,” were collected from Napo Cuizhuyuan Forestry Technology Co. LTD in Baise District, Guangxi, China (Figure 1A) and the field of Central South University of Forestry and Technology, Changsha, China, respectively. Samples were collected every 10 days from 200 to 240 day after full bloom for the variety of “Cenruan 2.” For the “3 Hua” series, the seeds were sampled at 265 days after full bloom for different genotype experiments. The horizontal and vertical diameters of “Cenruan 2” fruits and seeds were recorded as sampling standards.

### The Inoculation of Explants

The seeds were taken from fruit, thoroughly washed with tap water for 2 h, dipped into 75% (*v/v*) ethanol for 50 s, and washed three times with sterile distilled water. The treated seeds were then surface sterilized in 10% NaClO (*v/v*) for 15–20 min and washed six times with sterile distilled water. After carefully removing the seed coat, the zygotic embryonic axis (ZE) and cotyledon (COT) were separated as explants.

### Embryo Initiation and Proliferation

According to a previous study, MS medium (Murashige and Skoog) was chosen as the basal culture medium for *C. oleifera* somatic embryo induction (Hu et al., 2014). To induce embryogenic calli, ZE, or COT were placed in embryo initiation medium (E1) in a 90 × 15-mm Petri dish (20 explants per dish). Embryo initiation medium (E1): MS medium containing a combination of different concentrations of 2,4-D (0.75, 1.5, 2.25, or 3.0 mg/L) and TDZ (0, 0.3, 0.6, 0.9, or 1.2 mg/L) based on the orthogonal design method. The initiation medium was supplemented with 0.65% (*w/v*) agar and 3% sucrose, and the pH was adjusted to 5.6–5.8 before autoclaving at 121°C for 20 min. Unless mentioned, all cultures were incubated at 25 ± 1°C in continuous darkness with a relative humidity of 50–60%.

To record embryogenic callus rates, proliferation rates, and callus status, the induced calli were subcultured onto fresh E1 medium at an interval of 30 days. The cultures were examined weekly for tissue growth. Each treatment contained 60 explants with three replicates. To explore the genotype differences of SE in *C. oleifera*, cotyledons of the “3 Hua” series were inoculated on embryo initiation medium.

### Embryo Development in DSE and ISE

For DSE or ISE and differentiation, the induced embryogenic callus cultures were transferred from initiation medium (E1) to development medium (E2) and observed regularly to capture the developmental processes of somatic embryos under a stereomicroscope. The E2 medium was designed as MS medium containing a combination of different concentrations of 2,4-D (0.19, 0.38, or 0.57 mg/L) and TDZ (0, 0.05, 0.1, or 0.2 mg/L) based on the orthogonal design method. The cultures were consistently kept in the dark until cotyledonary embryos formed.

### Embryo Maturation and Germination

To observe the maturation of somatic embryos, the dehydration method by Muhedaner et al. (2019) was employed. Briefly, one sterile filter paper was added to the surface of MS medium without any phytohormones for somatic embryo maturation and incubated under a 16/8 h photoperiod/dark at 25 ± 1°C until green cotyledons formed. The mature cotyledonary embryos were cultured on embryo germination medium (E3), which was half-strength MS basal medium with different concentrations of IBA (0, 0.3, 0.6, 0.9, or 1.2 mg/L), to test the optimal germination conditions. A two-phase culture method was employed for the germination of mature somatic embryos. The mature cotyledonary embryos were first placed in a small tube with root polarity in contact with the surface of medium and then transferred to glass bottles with more room after the roots and shoots sprouted. The somatic embryos were incubated under a 16/8 h photoperiod/dark at 25 ± 1°C until an intact plant was formed. The development of roots and shoots was observed and regeneration rates were recorded.

### Morphological and Histological Observations

The different developmental stages of direct and indirect somatic embryos were observed at the tissue level and photographed under a stereomicroscope equipped with a Lycra color digital camera system (Lycra, M205FA, Germany). Paraffin sections were obtained for embryogenic tissues and embryogenic calli or non-embryogenic calli at 40 and 50 day after inoculation, respectively. For somatic embryo differentiation, the embryogenic calli at 70–85 days after inoculation were sectioned. The embedded procedures were as follows: fresh tissues at different developmental periods were sampled and immediately fixed in FAA (50% ethanol: glacial acetic acid: formaldehyde, 18:1:1). After fixing for 24 h, the tissues were dehydrated in an ethanolic-graded series (30, 50, 70, 90, and 100% v/v) sequentially for 60 min at each step. Tissue samples were embedded in paraffin wax, and 10-μm sections were sliced with a manual microtome. Sections were dewaxed with 0.5% safranine O and 0.1% fast green and examined under a light microscope by the method of Berlyn et al. (1976) with modification. Photographs were taken at different magnifications.

### Statistical Analysis

For SE, all of the trials were conducted with at least three replicates. The data were recorded using Excel 2016 software and analyzed by analysis of variance (ANOVA). Mean values were shown with standard errors. The significant differences were determined at the 1% level with Duncan’s multiple range tests using SPSS 21.0 software.

## Results

### Induction of Explants at Different Developmental Stages

**FIGURE 2:**
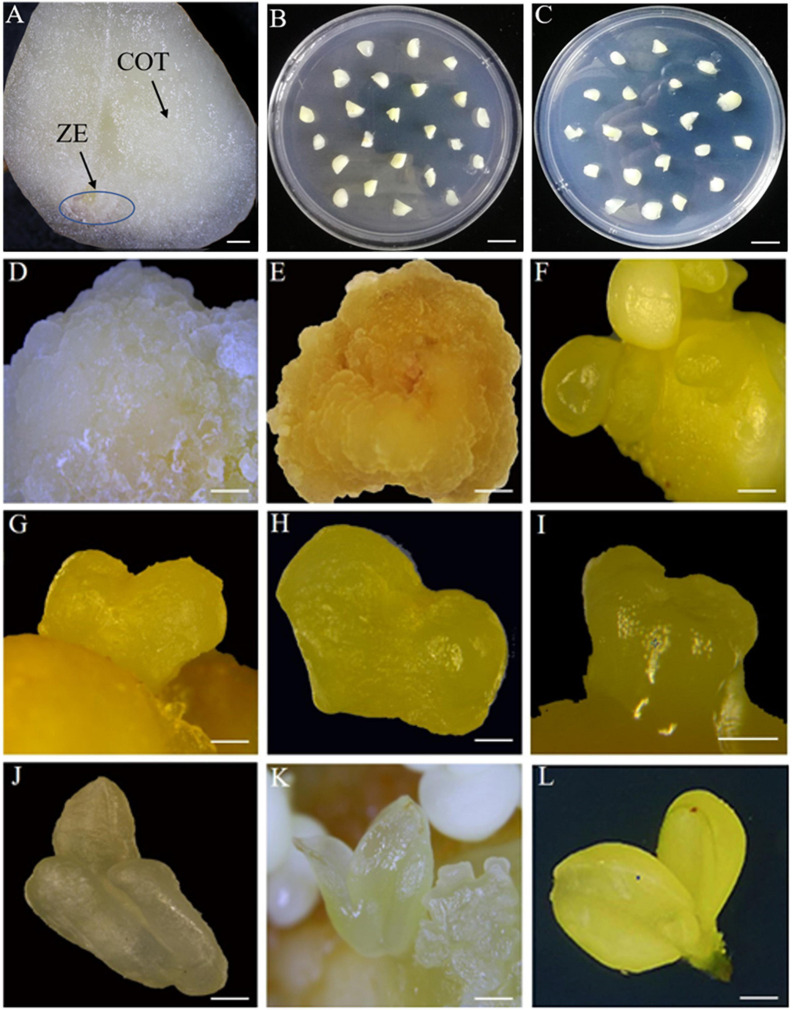
The observation of indirect somatic embryogenesis pathway at different developmental stages. **(A)** Zygotic embryonic axis (ZE) and cotyledons (COT) of immature seeds. **(B)** Zygotic embryonic axis inoculated in MS medium. **(C)** Cotyledons inoculated in MS medium. **(D)** Non-embryogenic calli 50 days after inoculation. **(E)** Embryogenic calli 50 days after inoculation. **(F)** Globular embryo 70 days after inoculation. **(G,H)** Heart embryo 75 days after inoculation. **(I,J)** Torpedo embryo 80 days after inoculation. **(K)** Cotyledonary embryo 85 days after inoculation. **(L)** Mature cotyledonary embryo 100 days after inoculation. Bars: **(A)** = 800 μm, **(B,C)** = 10 mm, **(D,E,L)** = 2 mm, **(I)** = 0.5 mm, and **(F,G,H,J,K)** = 1 mm.

The seeds collected at 200 day after blossoming were slightly white, gelatinous inside and soft in texture and then gradually turned yellow and hard (Figure 1B). With fruit development, the horizontal and vertical diameters of seeds also increased (Figures 1C,D). The ZE and COT of immature embryos at different developmental stages were cut and induced for embryonic calli (Figures 2A–C). Since the 200-day-old seeds still appeared as a liquid substance that resulted in failure to cut the corresponding part, 210-day-old to 240-day-old ZE or COT were employed and inoculated on medium for the induction of SE.

As shown in Table 1, the rates showed a “low-high-low” trend from 210–240 days after full- bloom. For 210-day-old ZE, 85 embryogenic callus clumps were induced with an induction rate of 16.7%, while 108 embryogenic callus clumps were obtained from 220-day-old ZE with a maximum induction rate of 40.9%. The induction rates of embryogenic calli further declined to 21.1 and 16.9% at 230 and 240 day, respectively. Compared to ZE, the numbers of callus clumps and embryogenic calli with COT as explants were relatively low; however, the embryogenic callus induction rates from 220-day-old ZE and COT showed no obvious differences, with rates of 40.9 and 39.3%, respectively (Table 1). Therefore, considering the availability of materials, COT from 220-day-old seeds was chosen as the explant for *C. oleifera* embryogenic callus induction.

**TABLE 1:** Embryogenic callus induction for *C. oleifera* immature seeds at different developmental stages.

**Sampling time**	**Explant types**	**Number of explants cultured**	**Number of callus clumps**	**Number of embryogenic callus clumps**	**Embryogenic callus induction rates (%)**
200 days	ZE COT	– –	– –	– –	– –
210 days	ZE COT	510 510	107 86	85 60	16.70 11.70
220 days	ZE COT	440 440	106 95	180 173	40.90 39.30
230 days	ZE COT	520 520	97 85	110 103	21.10 19.80
240 days	ZE COT	415 415	50 45	70 45	16.90 10.80

*“–” means the failure of explant cutting due to the liquid substance of seeds at this period.*

### Initiation and Proliferation of the Embryogenic Callus

**FIGURE 3:**
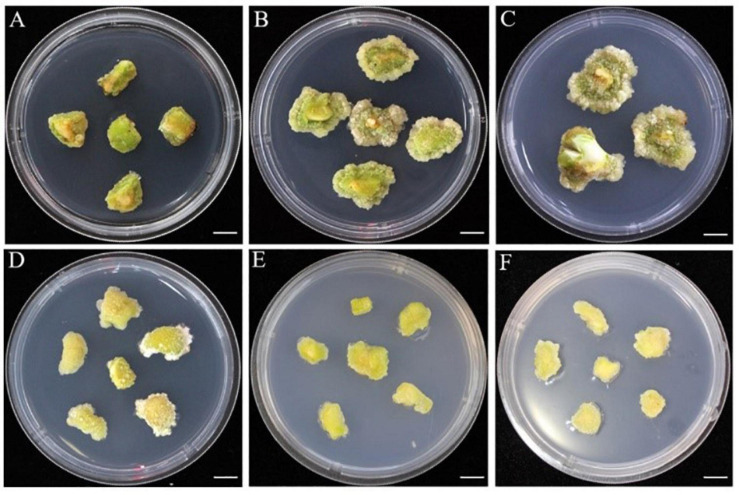
The phenotype of callus induced 50 days after inoculation under combined with 2,4-D and TDZ in E1 medium. **(A)** Induced callus status based on the treatment of E1-3, 5, 14, and 15. **(B)** Induced callus status based on E1-2 treatment. **(C)** Induced callus status based on treatment of E1-4. **(D)** Induced callus status based on treatment of E1-7, 8, 11, 12, 17, and 20. **(E)** Induced callus status based on treatment of E1-9, 18. **(F)** Induced callus status based on treatment of E1-16, 19, and 21. Bars: **(A–F)** = 10 mm.

Embryogenic callus induction is the first step in ISE. To explore the optimal induction conditions, we designed 21 combinations of different concentrations of 2,4-D with TDZ in E1 medium based on orthogonal design treatment for embryogenic callus induction (Table 2). After 40 days of inoculation, a small amount of induced embryogenic calli on explants appeared and the shape of explants still can be seen clearly, while a large amount of soft embryogenic calli were induced at 50 days after inoculation (Supplementary Figure 1). After 50 days of inoculation, two types of calli were induced from the immature cotyledons. Non-embryogenic calli appeared soft and milky white in E1-10 or E1-13 treatment (Table 2 and Figure 2D), while embryogenic calli appeared loose, light and brownish yellow in E1-1 or E1-6 treatment without TDZ (Table 2 and Figure 2E). The callus cultivated in other treatments of E1 medium failed to continue to develop in the later period, mostly compact, green or brownish yellow (Table 2 and Figure 3). We found that when the concentration of 2,4-D was relatively low, the induction rates of embryogenic calli decreased with increasing TDZ. For instance, the induction rates declined from 45.57 to 0.9% with increasing TDZ content when 2,4-D was 0.75 mg/L (Table 2). These results indicated that 2,4-D probably plays a dominant role during the process of *C. oleifera* embryogenic callus induction, while TDZ functions as a negative regulator to some extent. We also found that E1 medium containing 1.5 mg/L 2,4-D (E1-6) showed the highest induction rate of 87.2%, while basal medium supplemented with 0.75 mg/L 2,4-D (E1-1) resulted in the highest embryogenic callus proliferation ratio of 13.67% (Table 2). In addition, we explored the effect of different genotypes on embryogenic callus induction using the cv “3 Hua” series. The results showed that embryogenic calli were induced from the cotyledons of “HuaXin,” “Huashuo,” and “Huajin” in E1-6 medium supplemented with 1.5 mg/L 2,4-D (Supplementary Figures 2A,B) and the embryogenic callus induction rates showed no significant differences between different varieties (Supplementary Table 1).

**TABLE 2:** The induction and proliferation rates of *C. oleifera* embryogenic callus with the combination of different concentrations of 2,4-D and TDZ.

**Treatment**	**2,4-D (mg/L)**	**TDZ (mg/L)**	**Embryogenic callus induction rates (%)**	Proliferation rates* after 30 days in culture**	**Callus status**
E1-1	0.75	0	45.57 ± 0.43b	13.67 ± 0.46a	Loose, light and brownish yellow
E1-2	0.75	0.3	4.80 ± 0.06ij	4.57 ± 0.18f	Compact, green and white
E1-3	0.75	0.6	5.60 ± 0.06hi	2.67 ± 0.50gh	Compact, green
E1-4	0.75	0.9	1.73 ± 0.03np	1.17 ± 0.18j	Compact, green and white
E1-5	0.75	1.2	0.90 ± 0.06p	1.53 ± 0.15j	Compact, green
E1-6	1.5	0	87.20 ± 1.14a	5.63 ± 0.09de	Loose, light and brownish yellow
E1-7	1.5	0.3	13.67 ± 0.56e	7.77 ± 0.43c	Compact, brownish yellow
E1-8	1.5	0.6	2.87 ± 0.09lm	3.03 ± 0.12gh	Compact, brownish yellow and white
E1-9	1.5	0.9	25.33 ± 0.20d	7.77 ± 0.09c	Compact, green and brownish yellow
E1-10	1.5	1.2	1.20 ± 0.06p	4.87 ± 0.18ef	Loose, soft, milky white
E1-11	2.25	0	40.33 ± 0.49c	10.00 ± 0.17b	Compact, brownish yellow
E1-12	2.25	0.3	12.17 ± 0.35f	2.33 ± 0.09hi	Compact. brownish yellow
E1-13	2.25	0.6	13.50 ± 0.58e	5.90 ± 0.21d	Loose, soft, milky white
E1-14	2.25	0.9	2.37 ± 0.07mn	2.34 ± 0.09hi	Compact, green
E1-15	2.25	1.2	11.50 ± 0.29fg	2.67 ± 0.41gh	Compact, green
E1-16	3.0	0	6.30 ± 0.06h	4.90 ± 0.15ef	Loose, light yellow
E1-17	3.0	0.3	3.60 ± 0.06kl	1.53 ± 0.20j	Compact, brownish yellow
E1-18	3.0	0.6	3.80 ± 0.06kl	3.17 ± 0.18g	Compact, green and brownish yellow
E1-19	3.0	0.9	10.70 ± 0.80g	10.47 ± 0.43b	Loose, light yellow
E1-20	3.0	1.2	6.20 ± 0.06h	1.60 ± 0.06ij	Compact. brownish yellow
E1-21	0	0	4.30 ± 0.12jk	5.63 ± 0.09de	Loose, light yellow

*Proliferation rates are the ratios of final callus fresh weight biomass to original callus fresh weight biomass after 30 days of culture. **Data are means of three replicates ± standard errors. Means within the same column followed by different letters indicate significant differences at p < 0.01 by Duncan’s multiple range test.

### Development of Somatic Embryos for ISE

To unravel the effect of phytohormones on the morphological differentiation of embryogenic calli, we designed 13 combinations of different concentrations of 2,4-D with TDZ (E2 medium). The induced embryogenic calli in E1 medium were transferred to embryo development medium (E2) for morphological differentiation. Similar to the process of embryogenic callus induction, 2,4-D was also the dominant regulator in the process of globular embryo formation (Table 3). The medium containing 0.19 mg/L 2,4-D without TDZ (E2-1) reached the highest number of globular embryos at 61.33, and the number declined with increasing TDZ content. When the TDZ concentration rose to 0.2 mg/L (E2-4), the number of globular embryos decreased by approximately 85% relative to E2-1 treatment. A similar trend was observed in the differentiation process of heart-shaped and torpedo embryos, suggesting that 2,4-D, not TDZ, is suitable for *C. oleifera* embryogenic callus differentiation.

**TABLE 3:** Indirect somatic embryogenesis and differentiation rates of embryogenic callus with the combination of 2,4-D and TDZ in *C. oleifera*.

**Treatment**	**2,4-D (mg/L)**	**TDZ (mg/L)**	**Number of globular embryos**	**Number of heart embryos**	**Number of torpedo embryos**	**Number of cotyledonary embryos**	Cotyledonary embryos induction rates*** (%)
E2-1	0.19	0	61.33 ± 1.20a	15.33 ± 0.33b	10.33 ± 0.88b	5.33 ± 0.33b	8.69
E2-2	0.19	0.05	16.33 ± 0.88d	7.67 ± 0.88de	2.67 ± 0.33d	0.00 ± 0.00d	0
E2-3	0.19	0.1	13.33 ± 0.33e	4.67 ± 0.33f	0.00 ± 0.00e	0.00 ± 0.00d	0
E2-4	0.19	0.2	9.00 ± 0.58f	2.67 ± 0.33g	0.00 ± 0.00e	0.00 ± 0.00d	0
E2-5	0.38	0	24.67 ± 1.20c	12.00 ± 0.58c	8.33 ± 0.33c	2.33 ± 0.33c	9.44
E2-6	0.38	0.05	9.33 ± 0.33f	2.33 ± 0.33g	0.00 ± 0.00e	0.00 ± 0.00d	0
E2-7	0.38	0.1	17.67 ± 0.88d	7.00 ± 0.58e	2.67 ± 0.33d	0.00 ± 0.00d	0
E2-8	0.38	0.2	12.33 ± 1.45e	6.33 ± 0.88ef	0.00 ± 0.00e	0.00 ± 0.00d	0
E2-9	0.57	0	17.67 ± 0.88d	4.67 ± 0.88f	0.00 ± 0.00e	0.00 ± 0.00d	0
E2-10	0.57	0.05	5.33 ± 0.33g	1.33 ± 0.33g	0.00 ± 0.00e	0.00 ± 0.00d	0
E2-11	0.57	0.1	51.67 ± 0.88b	16.67 ± 0.88b	8.00 ± 0.58c	4.33 ± 1.20b	8.38
E2-12	0.57	0.2	15.00 ± 0.58de	7.33 ± 1.20e	2.33 ± 1.45d	0.00 ± 0.00d	0
E2-13	0	0	15.00 ± 0.58de	9.33 ± 0.33d	7.00 ± 0.58c	2.33 ± 0.33c	15.53
E2-13(1)*	0	0	62.00 ± 0.00**	52.67 ± 0.33a	45.00 ± 0.58a	38.67 ± 0.88a	62.37

*Represents the treatment in which globular embryos was transited into the phytohormones-free medium for the subsequent differentiation. **The initial number of globular embryos which was first allowed to form in E2-1 and then transferred to E2-13(1) is 62. ***Cotyledonary embryos induction rates are the ratios of final number of cotyledonary embryos to original number of globular embryos. Means within the same column followed by different letters indicate significant differences at p < 0.01 by Duncan’s multiple range test.

Distinct globular embryos occurred after 70 days of inoculation and then gradually converted into heart-shaped and torpedo embryos after 75 and 80 days, respectively (Figures 2F–J). After 85 days, embryo groups developed in the process of cotyledonary embryos, characterized by distinct bipolarity and easy separation from the mother callus (Figure 2K). Surprisingly, we found that an almost 75% loss of the induction rate occurred in the transition from globular embryos to heart embryos on medium supplemented with 0.19 mg/L 2,4-D (E2-1), which meant that E2-1 treatment was suitable for globular embryo formation but not for subsequent embryo differentiation. Therefore, we attempted to transfer these globular embryos formed on E2-1 medium in the first 20 days to E2-13(1) medium without any phytohormones and found that these embryogenic calluses produced an optimal number of cotyledonary embryos with an induction rate of 62.37% from globular embryos to cotyledonary embryos compared to E2-1 medium with only 8.69% (Table 3). Thus, it is obvious that 0.19 mg/L 2,4-D is suitable for globular embryo differentiation from somatic calli, and timely removal of phytohormones from the medium is also important for subsequent morphological differentiation. These results also suggest that globular embryo formation is the key process in ISE and that somatic embryo development and differentiation become natural developmental processes without requiring hormonal stimulation. Similar developmental processes of SE in the cv “3 hua” series were also observed (Supplementary Figures 1C–F), and no significant differences of cotyledonary embryos induction rates were found between different varieties (Supplementary Table 2).

### Development of Somatic Embryos for DSE

**FIGURE 4:**
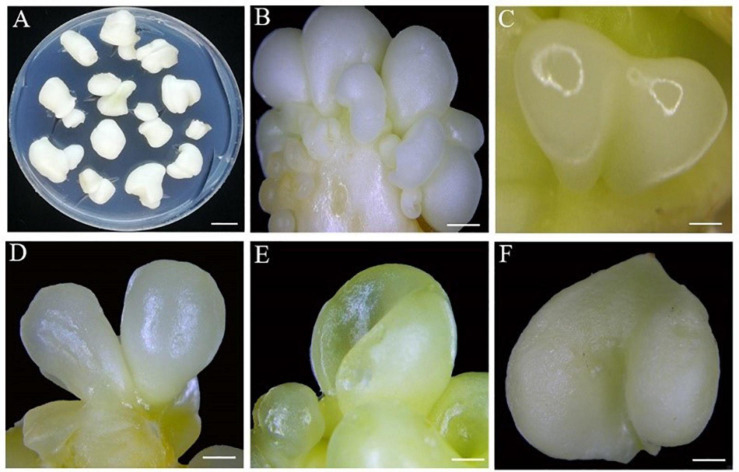
The observation of direct somatic embryogenesis pathway at different developmental stages. **(A)** 30 days after inoculation. **(B)** Globular embryo 50 days after inoculation. **(C)** Heart embryo 55 days after inoculation. **(D)** Torpedo embryo 60 days after inoculation. **(E)** Cotyledonary embryo 65 days after inoculation. **(F)** Mature cotyledonary embryo 80 days after inoculation. Bars: **(A)** = 2 mm, **(B–D)** = 1 mm, and **(E,F)** = 4 mm.

For DSE, the explants were also inoculated in E2 medium supplemented with different concentrations of 2,4-D and TDZ (Table 4). Unlike ISE, the combination of 0.19 mg/L 2,4-D and 0.05 mg/L TDZ (E2-2) showed a cotyledonary embryos induction rate of 53.96%, much higher than that at 0.19 mg/L 2,4-D (E2-1, 5.05%), suggesting that a certain ratio of 2,4-D and TDZ might play a significant role in the process of DSE. Furthermore, higher induction rates for subsequent heart embryos, torpedo embryos, and cotyledonary embryos were also obtained in E2-2 medium with numbers of 57.67, 43.33, and 36.33, respectively. These results indicated that phytohormones are necessary for the maintenance of DSE. For morphological observation, somatic embryos were directly induced from the surface of explants in E2-2 medium. After 30 days of inoculation, the explants became swollen and soft (Figure 4A), and globular embryos were observed after 50 days of culture, followed by heart embryos, torpedo embryos and cotyledonary embryos at 55, 60, and 65 day, respectively (Figures 4B–E). The induced cotyledonary embryos matured after 80 days of inoculation (Figure 4F).

**TABLE 4:** Direct somatic embryogenesis and differentiation from COT with the combination of 2,4-D and TDZ in *C. oleifera*.

**Treatment**	**2,4-D (mg/L)**	**TDZ (mg/L)**	**Number of globular embryos**	**Number of heart embryos**	**Number of torpedo embryos**	**Number of cotyledonary embryos**	Cotyledonary embryos induction rates* (%)
E2-1	0.19	0	26.33 ± 1.76b	9.67 ± 1.20cd	4.00 ± 0.58c	1.33 ± 0.33c	5.05
E2-2	0.19	0.05	67.33 ± 0.88a	57.67 ± 1.33a	43.33 ± 0.88a	36.33 ± 0.67a	53.96
E2-3	0.19	0.1	16.00 ± 2.08de	7.33 ± 1.20de	0.00 ± 0.00de	0.00 ± 0.00d	0
E2-4	0.19	0.2	16.33 ± 0.88d	7.33 ± 1.20de	3.33 ± 0.88cd	0.00 ± 0.00d	0
E2-5	0.38	0	26.67 ± 0.88b	14.67 ± 0.88b	6.67 ± 0.88b	1.67 ± 0.33c	6.26
E2-6	0.38	0.05	27.67 ± 1.45b	14.00 ± 1.15b	6.67 ± 0.88b	4.00 ± 0.58b	14.46
E2-7	0.38	0.1	6.33 ± 0.88f	1.33 ± 0.33f	0.00 ± 0.00de	0.00 ± 0.00d	0
E2-8	0.38	0.2	6.67 ± 1.20f	2.33 ± 0.33f	0.00 ± 0.00de	0.00 ± 0.00d	0
E2-9	0.57	0	6.33 ± 0.88f	0.00 ± 0.00f	0.00 ± 0.00de	0.00 ± 0.00d	0
E2-10	0.57	0.05	26.33 ± 0.88b	11.00 ± 1.53c	4.33 ± 0.67c	0.00 ± 0.00d	0
E2-11	0.57	0.1	21.00 ± 0.58c	7.67 ± 0.33de	1.00 ± 0.58de	0.00 ± 0.00d	0
E2-12	0.57	0.2	12.67 ± 1.20e	7.67 ± 0.33de	1.00 ± 0.58de	0.00 ± 0.00d	0
E2-13	0	0	13.33 ± 0.33de	6.00 ± 0.58e	1.67 ± 0.33de	0.00 ± 0.00d	0

*Cotyledonary embryos induction rates are the ratios of final number of cotyledonary embryos to original number of globular embryos. Means within the same column followed by different letters indicate significant differences at p < 0.01 by Duncan’s multiple range test.

### Histological Observation of SE

**FIGURE 5:**
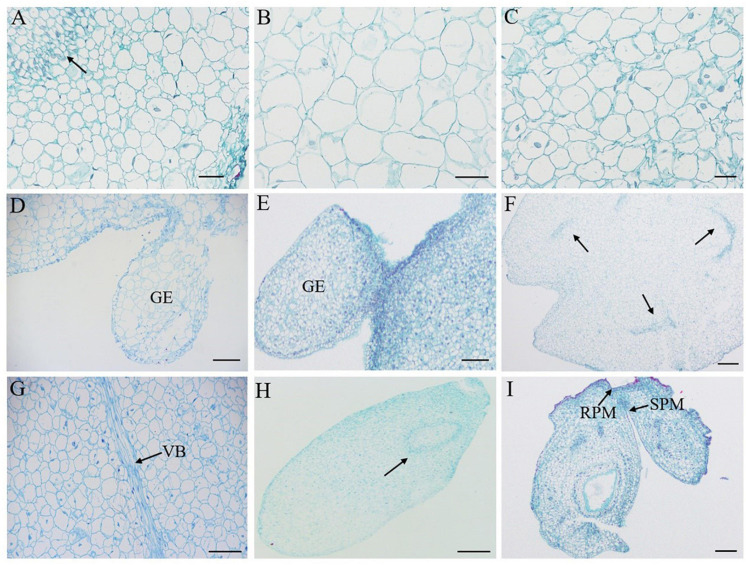
Histology observation of direct and indirect somatic embryogenesis from cotyledons of *C. oleifera.* **(A)** Embryogenic formation and aggregation 40 days after inoculation (arrow). **(B)** Non-embryogenic calli 50 days after inoculation. **(C)** Embryogenic calli at 50 days after inoculation. **(D)** Globular embryo at 70 days after inoculation in indirect somatic embryogenesis. **(E)** Globular embryo at 50 days after inoculation in direct somatic embryogenesis. **(F,G)** Transitional from globular to cotyledonary embryo with well-differentiated vascular bundle (VB) of somatic embryo (arrow). **(H)** Torpedo embryo with closed vascular bundle 80 days after inoculation. **(I)** Mature cotyledonary embryo with root and shoot pole meristems (RPM, SPM) 85 days after inoculation. Bars: **(A–C)** = 40 μm, **(D,G)** = 100 μm, and **(E,F,H,I)** = 200 μm.

Histological observation is an important guarantee and tool to prove the morphological differentiation and structure formation of somatic embryos. Therefore, in this study, induced somatic embryos of different developmental stages were embedded in paraffin wax and 10 μm sections were obtained for observation. As shown in Figure 5A, embryogenic tissues were observed 40 days after inoculation, showing mitotic division in cells. After 50 days of culture, two types of calli were observed. One was the embryogenic callus, which was composed of meristematic cells with dense cytoplasm and prominent nuclei, and the other was non-embryogenic callus, which was characterized by thin cytoplasm and rare nuclei (Figures 5B,C). Subsequently, the embryonic cells continued to differentiate, and globular embryos gradually formed after 70 days (Figure 5D). During DSE, the aggregation of embryonic cells directly formed somatic embryos (Figure 5E), which was different from ISE. The phenomenon of vascular bundle isolation from the mother tissue is believed to be an important indicator for the distinction between SE and organogenesis (Haensch, 2004; Lv and Shi, 2013). Here, we observed the obvious vascular bundle isolation of embryos from the mother tissue during the transition from globular embryo to cotyledonary embryo (Figures 5D–G), suggesting the reliability of SE in this study. After 80–85 days of inoculation, a closed vascular system was observed in the somatic embryos with shoot and root meristems (Figures 5H,I). These results confirmed that embryo development occurred via SE in *C. oleifera*.

### Maturation and Germination of Somatic Embryos

**FIGURE 6:**
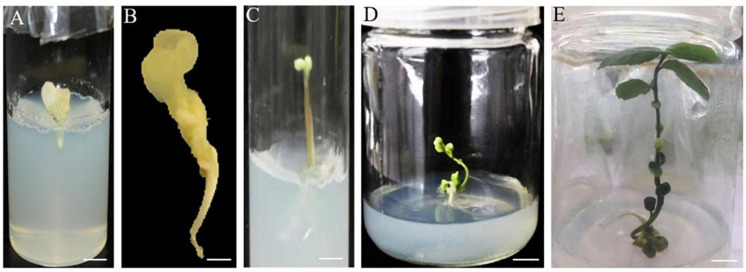
Plantlet regeneration in direct and indirect somatic embryogenesis of *C. oleifera*. **(A,B)** Regenerated roots 110–115 days after inoculation in indirect somatic embryogenesis. **(C)** Regenerated shoots 90 days after inoculation in direct somatic embryogenesis. **(D,E)** Plantlet with shoots and roots ready for acclimatization 120–130 days after inoculation. Bars: **(A,C,D,E)** = 1 mm and **(B)** = 0.5 mm.

When embryogenic calli developed into cotyledonary embryos, they were immediately transferred onto embryo maturation medium (MS basal medium). The culture lasted for 15 days until green cotyledons formed (Figures 2L, 4F). Microscopic observation showed that the mature cotyledon embryos had obvious root apex growth points (Figure 5I). The germination of somatic embryos is a major bottleneck for the regeneration of plants from SE. To explore suitable conditions for mature cotyledon germination, we designed IBA concentrations with different gradients on half-strength MS medium (E3 medium; Table 5). The results showed that the roots and shoots were obtained directly from phytohormone-free treatment after cotyledonary embryo germination (E3-1; Figure 6). With increasing IBA concentration, the germination rates significantly declined from the highest 63.67% (E3-1) to the lowest 4.33% (E3-5; Table 5). The induced somatic embryos germinated at 110–115 days after inoculation for ISE and 90 days for DSE. Leaves appeared fleshy and curly 20 days after germination (Figure 6D), and normal foliage occurred after another 10 days of continuous culture (Figure 6E).

**TABLE 5:** Germination of somatic embryos with different concentrations of IBA in medium.

**Treatment**	**IBA (mg/L)**	Regeneration rates* (%)
E3-1	0	63.67 ± 0.88a
E3-2	0.3	20.00 ± 1.15b
E3-3	0.6	13.33 ± 0.88c
E3-4	0.9	5.67 ± 1.76d
E3-5	1.2	4.33 ± 1.20d

*Regeneration rates are the ratios of intact plants with germinated roots and stems to inoculated cotyledonary embryos. Different letters indicate significant differences between treatments with different concentrations of IBA at p < 0.01.

## Discussion

Plant cells have the capacity for totipotent growth to produce a new plantlet through SE or organogenesis. SE can be induced *in vitro* by exposing a wide range of explants to suitable growth conditions (Yang and Zhang, 2010; Horstman et al., 2017). In recent years, SE has been employed in plant species as a propagation tool in place of seed reproduction (Ipekci and Gozukirmizi, 2003; Li et al., 2012), especially for plants with extremely low reproduction rates or maintaining agronomic traits. To date, the process and mechanism of SE in model plants such as *Arabidopsis* have been well elucidated (Kadokura et al., 2018; Wojcik et al., 2018); however, the widespread application of *in vitro* embryogenesis is limited to the low responsiveness of many plant species and genotypes, especially in woody plants.

The explants used for SE involve immature zygotic embryos or cotyledon, leaves, and floral tissues, etc. (Corredoira et al., 2019; Martínez et al., 2019). Although somatic embryos can be induced from the mature zygote embryos of adult plants (Silva-Cardoso et al., 2020), it seems to be more difficult for developmentally older tissues and organs to induce somatic embryos, especially for most woody plants. Compared with immature explants or embryonic tissues, more reprogramming is required in mature tissues to transform them into somatic embryos (Merkle et al., 1995). In this study, we chose COT and ZE from seeds 200–240 days after blossoming as explants and found that 220-day-old COT or ZE had the highest embryogenic callus induction rates, and the induction rates of 240-day-old explants declined by 24%, in both ZE and COT, indicating that explant age affected reprogramming and SE efficiency in *C. oleifera*. Actually, in Holm oak, callus induction was obtained only from explants collected at specific sampling time (Mauri and Manzanera, 2003). Shirin et al. (2020) found that in *Dalbergia latifolia*, somatic embryo formation was achieved from immature cotyledons 90 days after flowering. In addition, we also found that ZE and COT from 210-day-old after full-bloom showed high mortality, because they were more likely to become brown after inoculation (Table 1). These results suggest that explants in appropriate developmental stages and ages are particularly crucial for the induction of embryogenic calli. Moreover, we further investigated the effect of genotype on the induction rates of embryogenic calli. Four varieties of *C. oleifera* tested in this study showed no significant differences in the induction efficiency of somatic embryos based on the same medium, indicating that 2,4-D is generally suitable for *C. oleifera* embryogenic callus induction. Wang et al. (2016) also reported that genotype was not a key factor in somatic embryo induction in Sweetgum. However, in *Quercus acutissima* or *Betula platyphalla*, the induction frequency of somatic embryos was significantly different among different genotypes (Yang et al., 2020), suggesting that whether there is a genotype difference for embryogenic callus induction varies from species to species.

In the process of SE, ISE begins with the formation of embryogenic calli, the embryogenic cells have large and densely staining nuclei and nucleoli and are densely cytoplasmic with high metabolic activity (Jim’enez and Bangerth, 2001), whereas DSE is characterized by the absence of a callus formation phase, and the plantlet directly regenerates from the explants (Ikeuchi et al., 2013). Our results showed that calli could be induced with different compactness and color under the combinations of concentrations of 2,4-D and TDZ with ZE or COT as explants (Figure 3). Non-embryogenic calli appeared soft and milky white, while embryogenic calli appeared loose, light and brownish yellow. The high induction and proliferation ratio for embryogenic calli was 0.75 or 1.5 mg/L 2,4-D without TDZ (Table 2), suggesting that 2,4-D plays a dominant role in the process of *C. oleifera* embryogenic callus induction or proliferation, while TDZ functions as a negative regulator. Embryogenic callus formation is derived from the development of proembryogenic masses on the surface or within the callus mass, from which single cells or cell clusters develop into embryos (Halperin, 1966). In many plants, auxin promotes callus and proembryogenic mass initiation and proliferation. Embryo initiation was mainly caused by the irregular distribution of auxin (Márquez-López et al., 2018). After applying 2,4-D in *Oryza sativa*, an embryogenic callus was successfully induced from mature seeds (Ahmad et al., 2013). Here, we also found that in *C. oleifera*, 2,4-D alone also promoted embryogenic callus formation, while combination with TDZ greatly hindered the formation of embryonal cells. A similar phenomenon was also described in *Musa acuminate* using single 2,4-D treatment (Rosa et al., 2014), which may be related to the dominant effect of the stress genes induced by 2,4-D on cellular reprogramming of the somatic cells toward embryogenesis (Kitamiya et al., 2000).

Phytohormones act either synergistically or antagonistically in different stages of SE (Kumar et al., 2020). Auxin and cytokinin can act together to promote the initiation of somatic embryos (Yang and Zhang, 2010). In our study, we observed that embryogenic calli and proembryogenic masses formed at 50 days after inoculation and that globular embryos differentiated at 70 days, followed by heart embryos, torpedo embryos and cotyledonary embryos at 75, 80, and 85 days, respectively (Figure 2). For DSE, the treatment with the combination of 2,4-D and TDZ showed a much higher induction rate of globular embryos than the application of 2,4-D alone, suggesting that a certain ratio of 2,4-D and TDZ might play a significant role in the process of DSE and that phytohormones are necessary for the maintenance of DSE in *C. oleifera*. Such combined favorable influence of auxin and cytokinins is in accordance with reports in *Malaxis densiflora* (Mahendran and Bai, 2016), date palm (Baharan and Mohammadi, 2018), and *Coelogyne cristata* (Naing et al., 2011). Conversely, the addition of 2,4-D alone was not favorable for DSE in *Oncidium* (Chen et al., 1999). It has been reported that a defined medium supplement with low dosages of TDZ can directly promote epidermal cells to form somatic embryos in *Oncidium* (Chen et al., 1999). In contrast, a medium without exogenous phytohormones showed an important impact on the subsequent differentiation of globular embryo formation during ISE in *C. oleifera* (Tale 3). Similarly, in Chinese chestnut, embryogenic calli were induced with 1.8 μM 2,4-D and 1.1 μM 6-BA, while a subsequent differentiation was accomplished on phytohormone-free medium (Lu et al., 2017). Kim et al. (2012) found that embryogenic calli from leaf explants of *S. mukorossi* yielded somatic embryos removing auxin and cytokinin. This suggested that auxin in the SE process was not persistent and was inhibited for the development of somatic embryos (Nomura, 1995). However, there are different reports in other species. In *M. koenigii*, phytohormones simultaneously determined the occurrence of two pathways. Embryogenic callus formation was achieved with 6-BA and NAA treatment, and globular somatic embryos and subsequent differentiation were induced on medium containing TDZ (Paul et al., 2011). These results demonstrated that the mechanism of SE is divergent among different species and that the degree of dependence on hormones is different for ISE and DSE.

As a heritable epiregulatory mechanism that inhibits gene expression, DNA methylation is found at different degrees in different stages of SE (Hao and Deng, 2002). In SE of carrots, removal of auxin resulted in reduced methylation followed by embryo development (Loschiavo et al., 1989). Likewise, the decrease in auxin levels allowed heart-shaped and torpedo embryos to continue to develop in *Cucurbita pepo*, accompanied by a significant decrease in DNA methylation (Leljak-Levanic et al., 2004). Our results also showed that the medium supplemented with 0.19 mg/L 2,4-D caused considerable loss of heart embryos (Table 3), which revealed that it was more conducive to reducing the concentration of 2,4-D for the development of somatic embryos and cotyledon embryo formation on phytohormone-free media. Therefore, auxin, especially 2,4-D, helps to promote the formation of embryogenic calli from explants and the development of proembryogenic masses in the ISE process of *C. oleifera*; however, the subsequent differentiation process is more similar to an endogenous phytohormones-driven process.

The conversion of somatic embryos into plantlets is also a limiting step in many woody plant species. The lack of maturation and desiccation tolerance is believed to be one of the factors that results in low plant recovery rates (Etienne et al., 1993). Polyethylene glycol (PEG) and abscisic acid (ABA) are usually used to promote the maturation of somatic embryos (Vale et al., 2018). In this study, the dehydration method by Muhedaner et al. (2019) was employed to promote the maturation of cotyledon embryos. Thus, there was no need to go through complicated concentration screening and avoid the slight toxic effect of the additive itself on the tissue. Recent evidence in *Musa acuminata* (Remakanthan et al., 2014) and *Picea schrenkiana* (Muhedaner et al., 2019) revealed that the presence of filter paper played an essential role in the desiccation of SE. However, desiccation with filter papers in oil palm caused excessive water loss, and ultimately, the maturation of somatic embryos was promoted by a balanced concentration of 25 μM ABA (Mariani, 2014); however, the early germination of somatic embryos was prevented by exogenous application of ABA in *E. camaldulensis* (Prakash and Gurumurthi, 2010).

**FIGURE 7:**
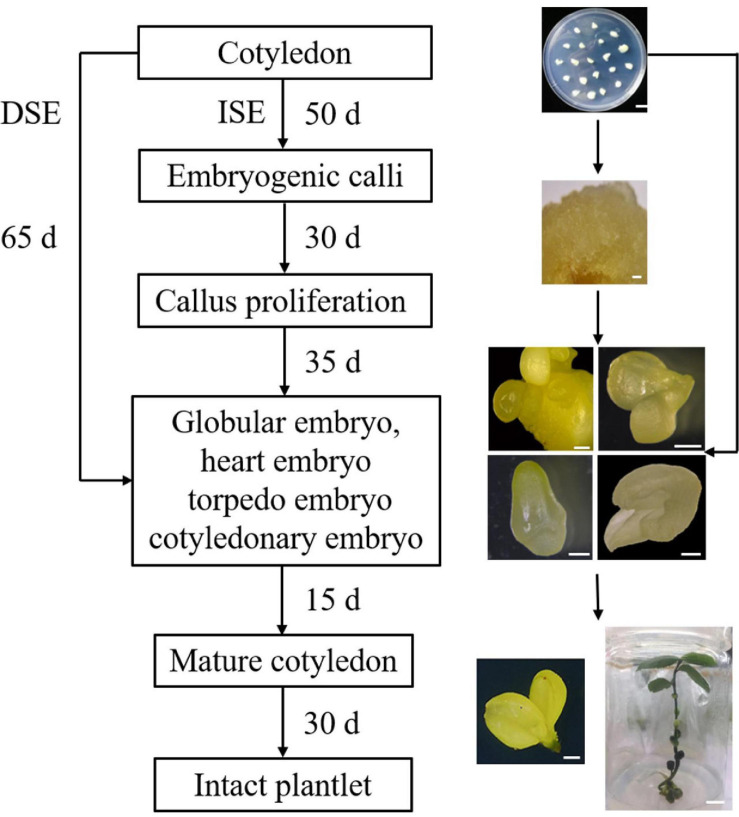
Flow chart for direct and indirect somatic embryogenesis regeneration system in *C. oleifera*.

Similar to zygotic embryos, *in vitro* somatic embryos are bipolar structures with an apical pole that develops into the shoot and a basal pole that develops into the future root. Each of these bipolar structures has its own meristem and an independent provascular system distinguishing SE from organogenesis (Horstman et al., 2017). In organogenesis, ectopic or adventitious organs, such as shoots and roots, are unipolar structures with a lignified vascular connection to the mother explants (Horstman et al., 2017). Therefore, whether morphogenesis originated from embryogenesis or organogenesis should be evaluated at the histological level. In this study, we observed the obvious vascular bundle isolation of embryos from the mother tissue during the transition from globular embryos to cotyledonary embryos (Figure 5). After 80–85 days of inoculation, a closed vascular system was observed in the somatic embryos with shoot and root pole structures. These results confirmed that embryo development in this study occurred via SE, not organogenesis. Actually, in *Camellia nitidissima*, the regenerated plantlets could initiate embryogenic callus clumps, whether by SE or by shoot organogenesis, depending on the phytohormones used in the medium (Lü et al., 2013). In a previous report of *C. oleifera*, embryonic calli regenerated plantlets by clumping buds and adventitious roots (Zhang, 2005), presenting a pattern of organogenesis for plantlet formation. Nevertheless, there is no doubt that SE is more conducive to industrialized seedling culture than the organogenesis pathway, especially in the application of artificial seeds. In *C. oleifera*, the inoculated explants were cultured for 65 days to differentiate into cotyledon embryos via the DSE pathway, and 85 days via the ISE pathway (Figure 7). Compared to ISE, DSE can be completed directly from the surface of explants, which requires minimal genetic reprogramming and a relatively short culture time (Arnold et al., 2002); thus, the DSE pathway can be applied to plant genetic transformation. However, the ISE pathway undergoes embryogenic callus induction and proliferation steps that are more suitable for mass production and industrialized seedling culture.

## Conclusion

In conclusion, we established a high-efficiency induction system for DSE and ISE in *C. oleifera* and achieved regenerated intact plantlets via SE, not organogenesis. For ISE, 2,4-D alone promoted embryogenic callus formation from explants and the development of globular embryos, while the subsequent differentiation was similar to an endogenous phytohormones-driven process. For DSEs, the combination of 2,4-D and TDZ improved the differentiation process, and phytohormones were consistently necessary for the maintenance of DSEs. Mature embryos germinated to produce intact plantlets on half-strength MS basal medium with a regeneration rate of 63.67%. In addition, there was no genotype difference for somatic embryo induction among the different varieties of *C. oleifera*. The improved SE protocol we provided would benefit industrialized seedling propagation and promote genetic manipulation of *C. oleifera* in the future.

## Data Availability Statement

The original contributions presented in the study are included in the article/Supplementary Material, further inquiries can be directed to the corresponding author/s.

## Author Contributions

LZ, YS, and MZ designed the experiments. MZ and AW carried out the experiments and analyzed the data. MQ, XQ, and SY contributed to the material treatment of cv “Cenruan” and photo processing. LZ, MZ, and YS contributed to the manuscript written. SS helped to edit the manuscript. All authors reviewed and approved the manuscript.

## Conflict of Interest

The authors declare that the research was conducted in the absence of any commercial or financial relationships that could be construed as a potential conflict of interest.

## Supplementary Material

Supplementary Figure 1 The observation of indirect somatic embryogenesis for cv “Cenruan 2” during embryogenic callus induction stages. **(A)** The explants 40 days after inoculation, showing the shape of explants and a small amount of induced embryogenic calli on it. **(B)** 50 days after explants inoculation, showing the soft embryogenic calli. **(C)** Enlarged image of the stripped embryogenic calli in (**B**; marked in red square) under stereo microscope. Bars** =** 10 mm in **(A–C)**.

Supplementary Figure 2 The observation of indirect somatic embryogenesis for the variety of “Huashuo” at different developmental stages. **(A)** Cotyledons inoculated in medium. **(B)** Embryogenic calli 50 days after inoculation. **(C)** Globular embryo 70 days after inoculation. **(D)** Heart embryo 75 days after inoculation. **(E)** Torpedo embryo 80 days after inoculation. **(F)** Cotyledonary embryo 85 days after inoculation. **(G)** Plantlet with shoots and roots ready for acclimatization 120 days after inoculation. Bars: **(A,B,G)** = 10 mm, **(C,E)** = 0.5 mm, and **(D,F)** = 1 mm.
