# Supplementary figures and images for: Direct and Indirect Somatic Embryogenesis Induction in Camellia oleifera Abel
Source: Front Plant Sci. 2021 Mar 26;12:644389. doi: 10.3389/fpls.2021.644389 (PMC8034400; doi:10.3389/fpls.2021.644389)

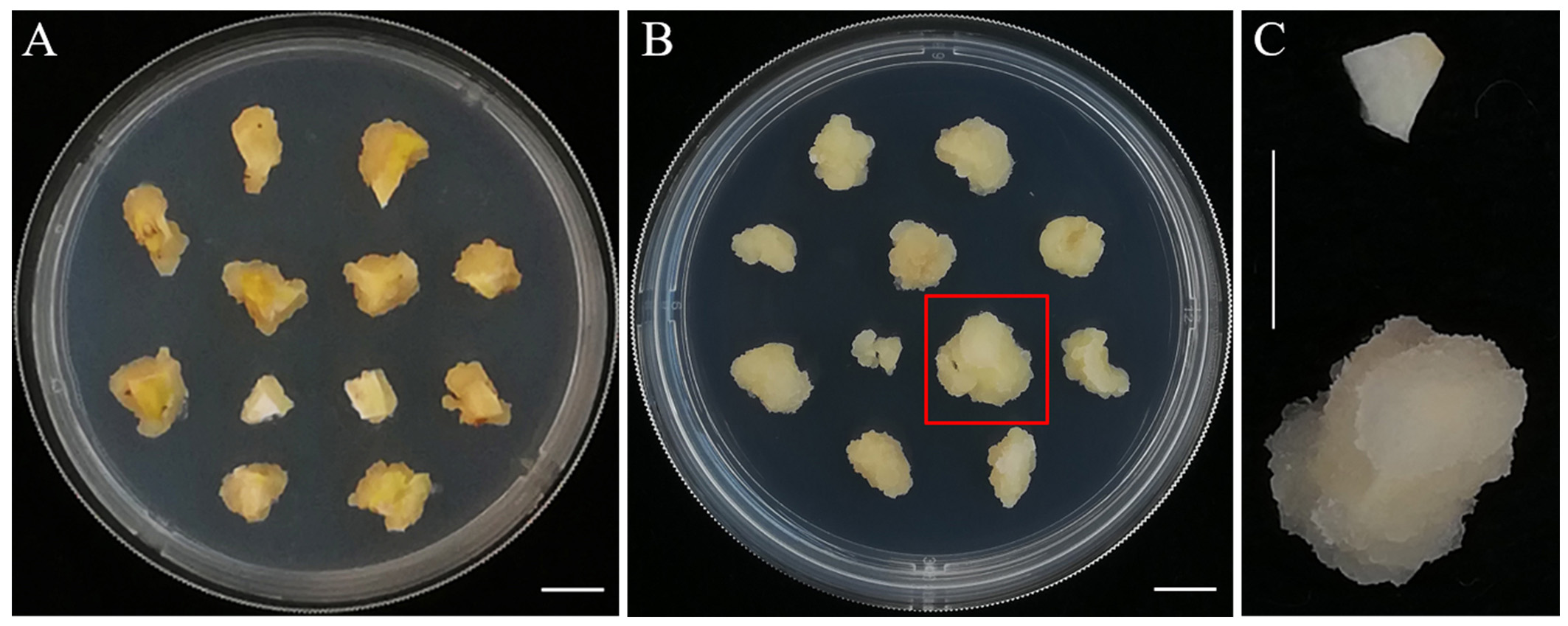

Supplement: Supplementary Figure 1 — The observation of indirect somatic embryogenesis for cv “Cenruan 2” during embryogenic callus induction stages. (A) The explants 40 days after inoculation, showing the shape of explants and a small amount of induced embryogenic calli on it. (B) 50 days after explants inoculation, showing the soft embryogenic calli. (C) Enlarged image of the stripped embryogenic calli in (B; marked in red square) under stereo microscope. Bars = 10 mm in (A–C). [file Image_1.JPEG]

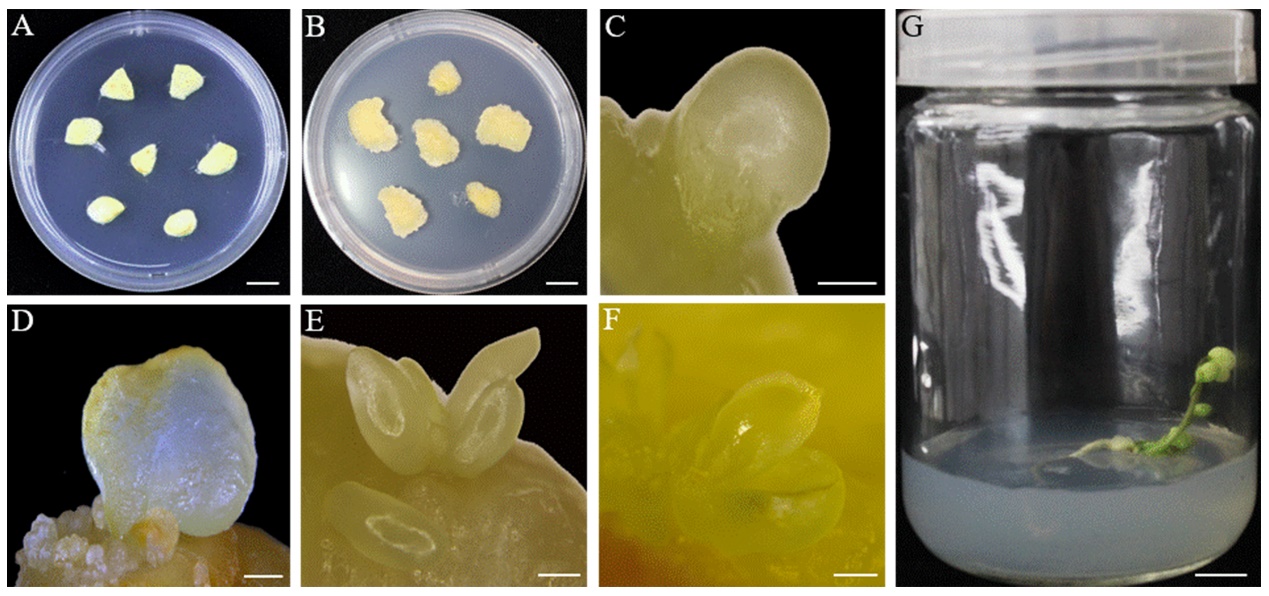

Supplement: Supplementary Figure 2 — The observation of indirect somatic embryogenesis for the variety of “Huashuo” at different developmental stages. (A) Cotyledons inoculated in medium. (B) Embryogenic calli 50 days after inoculation. (C) Globular embryo 70 days after inoculation. (D) Heart embryo 75 days after inoculation. (E) Torpedo embryo 80 days after inoculation. (F) Cotyledonary embryo 85 days after inoculation. (G) Plantlet with shoots and roots ready for acclimatization 120 days after inoculation. Bars: (A,B,G) = 10 mm, (C,E) = 0.5 mm, and (D,F) = 1 mm. [file Image_2.JPEG]
