# Supplementary material for: Direct and Indirect Somatic Embryogenesis Induction in Camellia oleifera Abel
Source: Front Plant Sci. 2021 Mar 26;12:644389. doi: 10.3389/fpls.2021.644389 (PMC8034400; doi:10.3389/fpls.2021.644389)
Supplement: Supplementary file 3 [file Table_1.DOCX]

Supplementary Material

**TABLE S1 | Embryogenic callus induction for different varieties after 50 days of culture in MS medium supplemented with 1.5 mg/L 2,4-D using cotyledons as explants.**

| Variety | Number of explants  cultured | Number of embryogenic  callus clumps | Embryogenic callus  induction rates (%) |
| --- | --- | --- | --- |
| Cenruan 2 | 200 | 174 | 86.67±0.01a |
| Huashuo | 200 | 169 | 84.50±0.12a |
| Huaxin | 200 | 172 | 86.17±0.12a |
| Huajin | 200 | 166 | 83.00±0.20a |

**TABLE S2 | Indirect somatic embryogenesis and differentiation rates of for different varieties**

| Variety | Number of globular embryos | Number of cotyledonary embryos | Cotyledonary embryos induction rates^*^ (%) |
| --- | --- | --- | --- |
| Cenruan 2 | 59.33±1.45a | 36.33±1.45a | 61.19±0.01a |
| Huashuo | 51.33±1.20a | 28.67±1.76ab | 55.91±0.04a |
| Huaxin | 54.00±0.58a | 27.33±1.76b | 50.67±0.04a |
| Huajin | 52.00±1.72a | 27.00±0.58b | 52.00±0.01a |

^*^*Cotyledonary embryos induction rates are the ratios of final* *number of cotyledonary embryos to original number of globular embryos.*
